# The role of MicroRNAs expression in laryngeal cancer

**DOI:** 10.18632/oncotarget.4195

**Published:** 2015-06-05

**Authors:** Xin Yu, Zheng Li

**Affiliations:** ^1^ Department of Orthopaedic Surgery, Peking Union Medical College Hospital, Chinese Academy of Medical Sciences and Peking Union Medical College, Beijing, China

**Keywords:** laryngeal cancer, miRNAs, oncogene, tumor suppressor gene

## Abstract

MicroRNAs (miRs, miRs) is a class of small non-coding RNAs, which posttranscriptionally regulate gene expression. Deregulated miRs are frequently obseved in patients with laryngeal cancer. In addition, numerous studies have showed miRs play significant roles in the pathogenesis of laryngeal cancer through regulating tumor cell proliferation, metastasis, invasion and apoptosis. miR can play either an oncogenic or tumor suppressive role in laryngeal cancer. In our review, we summarize the recent researches on laryngeal cancer-associated miRs, focusing on their role in the pathogenesis of laryngeal cancer. As changes in the levels of specific miRs in tissues or serum associate with diagnosis and prognosis of patients, we will also discuss the potential use of miRs in laryngeal cancer diagnosis and prognosis. Furthermore, supplementation of oncomiRs or inhibition of tumor suppressive miRs *in vivo* may be future therapeutic strategy for laryngeal cancer.

## INTRODUCTION

MicroRNAs (miRNAs or miRs) are a class of non-coding RNAs with approximately 22-nucleotides [1–7]. miRs posttranscriptionally modulate gene expression by binding to messenger RNA, leading to transcript degradation or translational inhibition. It is well known that miRs play significant roles in various human cancers, functioning as oncogenes or tumor suppressor genes [1, 8–21]. Laryngeal cancer is the eleventh most common cancer worldwide with high mortality rate. Laryngeal squamous cell carcinoma (LSCC) accounts for approximately 85–90% of all malignant tumors of the larynx [22–24]. Despite therapeutic advances in recent years, the clinical outcome for patients with advanced laryngeal cancer has not improved in the last 20 years [25–27]. This is mainly because of the lack of definite diagnosis, leading to reduced treatment efficacy and frequent recurrent rate. In addition, resistance to chemotherapy or radiotherapy and high local or regional recurrent rate after surgery also account for the high mortality. Thus, it is urgent to better understand the underlying molecular mechanisms and to develop more effective diagnostic and therapeutic targets. The recently discovered microRNAs (miRs or miRs) have been implicated in the development of laryngeal cancer.

In this review, we will discuss some significant miRs involved in laryngeal cancer development, focusing on their roles as oncogenes or tumor suppressors. In addition, we will also include their potential implications in the creation of novel diagnostic and prognostic tools or promising future therapies.

### MicroRNAs and cancer

miRs play crucial roles in many physiological and pathological processes through regulating cellular proliferation, differentiation, metabolism and apoptosis [2, 13, 17, 28, 29]. One miR can regulate the expression of multiple mRNAs and different miRs can modulate an individual mRNA. It is considered that miRs can regulate approximately 60% of genes in the human genome. Abnormal miRs expression is involved in many human diseases, especially cancers [30–32]. miR microarrays have demonstrated that abnormal miRs expression may function as potential diagnostic and prognostic biomarkers for cancers. In addition, miRs play significant roles in the pathogenesis of cancers, acting as oncogenes, tumor suppressors, or modulators of cancer stem cells [9, 33]. miRs may act as oncogenes by inhibiting tumor suppressor genes and act as tumor suppressor genes by downregulating oncogenes. For example, miR-221 and miR-222 act as oncogenes by inhibiting the expression of tumor suppressor gene: p27Kip. Based on these discoveries, miR-based therapeutic methods have been developed.

### Dysregulation of MicroRNAs in laryngeal cancer

Evaluation of miR expression by large-scale microarray analysis has demonstrated numerous miRs changes in laryngeal cancer [34, 35]. It can be used to compare differential miR expression between normal and cancer tissues, primary lesions and metastatic/recurrent lesions, or miR expression differences induced by anticancer treatment. In addition, quantitative RT- PCR is often used in validating miR expression in clinical specimens. Recent studies have identified many miRs with cancer-specific upregulation (oncomiRs) or downregulation (tumor suppressor miRs) [36]. As far as concerned, there have been 6 miR expression profiles of laryngeal cancer tissues and paired control tissues. In addition, the expression profiles of miRs was also analyzed in plasma from laryngeal squamous cell carcinoma (LSCC) patients and healthy controls.

Cao et al. compared the different miR expression profiles between 6 pairs of laryngeal SCC and the surrounding normal tissues using miR array. They showed 26 upregulated miRs and 3 downregulated miRs. Among them, miR-21, miR-93, miR-205, and miR-708 were upregulated while miR-125b and miR-145 were downregualed validated by quantitative RT-PCR [35].

Another study examined different miR expression profiles between laryngeal carcinoma tissue and adjacent normal tissue specimens using both microarray and quantitative real-time polymerase chain-reaction analyses. They showed that two miRs (miR-21-3p and miR-106b- 3p) was upregulated and six miRs (let-7f-5p, miR-10a-5p, miR-125a-5p, miR-144-3p, miR-195-5p, and miR-203) was downregulated [37].

Using miR microarray technique and bioinformatic algorithms to identify biomarkers for early diagnosis of larynx carcinoma, 47 miRs were differentially expressed in primary larynx tumor tissues compared to normal tissues. Of these, 30 were upregulated and 17 downregulated. In addition, overexpression of hsa-miR-657 and downexpression of hsa-miR-1287 demonstrated high sensitivity and specificity for discriminating between early larynx carcinoma and normal mucosa tissues [38].

Saito et al. reported the expression profiles of 723 human mature miRs in 5 laryngeal tissues using microarrays. The expression of 5 miRs (miR-130b-5p, miR-196a, miR-455-3p, miR-455-5p, and miR-801) was significantly upregulated whereas 2 miRs (miR-133b and miR-145) were downregulated in laryngeal cancers [39].

In a study to analyze the miRs expression associated with epithelial-mesenchymal transition (EMT) in lymph node metastasis of LSCC, miRNA microarray gene-expression profiling was performed to identify the differences between lymph nodes metastasis N (+) and lymph nodes metastasis-free (N0) groups. Nine miRNAs were upregulated and one miRNA was downregulaed in N (+) group compared with N0 group. Furthermore, the genes for miR-192, miR-143, miR-409 and miR-634 could promote EMT of LC cells through targeting Krüppel-like factor 17(KLF17), E-cadherin and phosphatidylinositol 3 kinase (PI3K). In addition, the miRNAs increased in group N(+) can be used to predict cervical lymph node metastasis in SGLSCC [40].

The expression profiles of 738 miRs in plasma from laryngeal squamous cell carcinoma (LSCC) patients and healthy controls were demonstrated using high-throughput real-time quantitative polymerase chain reaction. Seventeen miRs were upregulated and nine miRs were downregulated in LSCC patients compared with the healthy controls. They also found that 5 miRs (miR-331-3p, 603, 1303, 660-5p and 212-3p) are LSCC specific and never seen before in plasma of any human subject [41] (Table 1).

**Table 1:** MiRNA expression profiles in laryngeal cancer

Reference	miRNA	Up- or downregulated
35	miR-21, miR-93, miR-205, and miR-708	Up
	miR-125b and miR-145	Down
37	miR-21-3p and miR-106b-3p	Up
	let-7f-5p, miR-10a-5p, miR-125a-5p, miR-144-3p, miR-195-5p, and miR-203	Down
38	hsa-miR-657	Up
	hsa-miR-1287	Down
40	miR-331-3p, 603, 1303, 660-5p and 212-3p	Up
41	miR-192, miR-143, miR-409 and miR-634	Up

### OncomiRs in laryngeal cancer

Many studies have examined the oncogenic roles of miRs in laryngeal cancer. miR microarray showed that miR-16 was overexpressed in laryngeal cancer tissues. When miR-16 was blocked in HEp-2 laryngeal cancer cell line, the cell adhesion capability was enhanced and cell migration was suppressed. Therefore, miR-16 acted as an oncogene in LSCC via down-regulation of Zyxin in laryngeal cancer cells [42]. Wu et al. demonstrated that miR-19a expression was significantly higher in LSCC tissue. *In vitro* analysis demonstrated that inhibition of miR-19a reduced proliferation and promoted apoptosis in LSCC cells. Furthermore, growth of LSCC xenograft tumors was significantly reduced by repeated injection of antisense oligonucleotides (ASO)-miR-19a lentivirus, which could inhibit the expression of miR-19a [43]. Liu et al. reported that miR-21 was upregulated in laryngeal carcinoma tissues and negatively regulated BTG2, which is a known pan-cell cycle regulator and tumor suppressor. Furthermore, overexpression of miR-21 enhanced tumor cell growth activity [44–47]. Tian et al showed that miR-27a was upregulated in the LSCC tissues compared to the adjacent non-tumor tissues. PLK2 expression level showed a negative correlation with miR-27a expression level. Both miR-27a and knockdown of PLK2 promoted proliferation and suppresses apoptosis in the Hep2 cells. Thus, miR-27a functioned as an oncogene in LSCC through targeting PLK2 [48]. In addition, Xu et al. demonstrated that miR-106b could increase the proliferation and invasion of laryngeal carcinoma cells of through targeting RUNX3 [49]. Downregulation of miR-129-5p inhibited cell proliferation and migration, and induced cell cycle arrest and apoptosis in Hep-2 cell lines. Moreover, miR-129-5p antisense oligonucleotides (ASO) in BALB/c mice showed a marked inhibition of the growth of LSCC xenograft. In addition, miR-129-5p was upregulated in primary LSCC tumors and adenomatous polyposis coli (APC) was identified as its direct target. In conclusion, miR-129-5p functioned as an oncogene in LSCC by repressing APC [50]. miR- 155 in LSCC was also found to be upregulated in LSCC tissues. In addition, miR-155 enhanced proliferation and invasion of LSCC by directly targeting and suppressing the expression suppressor of cytokine signaling 1 (SOCS1) and STAT3 [51]. Downregulation of miR-196a inhibited cancer cell proliferation in both laryngeal cancer-derived cells and orthotopic xenograft model in mice. Therefore, miR-196a could strongly promote laryngeal cancer growth and the inhibition of miR-196a effective to reduce the *in vivo* growth of LSCC [39]. Li et al. demonstrated overexpression of miR-1297 in LSCC and Hep-2 cells. Downregulation of miR-1297 in Hep-2 cells inhibited cancer cell proliferation, migration, and tumor genesis in LSCC. In addition, PTEN, a well-known tumor suppressor gene downregulated in a variety of cancers, was identified as a direct target of miR- 1297 [52, 53].

### Tumor suppressor MicroRNAs in laryngeal cancer

Many tumor suppressor miRs have also been investigated in laryngeal cancer. For example, miR-1 inhibited growth, migration and invasion in the HEp2 cell line via directly targeting FN1. In addition, the expression levels of miR-1 were higher in LSCC tissues than that in adjacent normal tissues. It was concluded that miR-1 might function as a tumor suppressor gene in laryngeal carcinoma [54]. Yan Guo found that miR-24 significantly induced cell morphology changes and inhibited cell proliferation and invasion ability in Hep2 cells vitro through down-regulation of S100A8, indicating that miR-24 may function as a tumor suppressor in LSCC by inhibiting S100A8 [55]. miR-30b was involved in regulation of p53, one important tumor suppressor gene. In miR-30b overexpressed HEp-2 cells, p53 expression was significantly enhanced. In addition, miR-30b overexpression increased p53-mediated tumor cell apoptosis both *in vitro* and *in vivo*. Therefore, miR-30b is a tumor suppressor miR [56]. miR-34a inhibited cell proliferation by arresting cells at G0/G1 through targeting survivin. In addition, In LSCC tissues, miR-34a was downregulated, inversely correlated with histologic differentiation and positively correlated with survival rate. These results indicated that miR-34a might function as a tumor suppressor in LSCC by targeting survivin [57]. Hsa-miR-34c inhibited growth and invasion of human laryngeal carcinoma cells via targeting and inhibiting c-Met, which was a proto-oncogene. Aberrantly active c-Met could trigger tumor growth. In human laryngeal carcinoma tissues, c-Met expression was higher and hsa-miR-34c expression was significantly lower than its paired normal tissues. Thus, these findings suggested that hsa-miR-34c might also be a tumor suppressor miR in laryngeal cancer. A recent study demonstrated that miR-126 could inhibit LSCC partly by targeting Camsap1 [58]. Luo et al. showed that miR-139 suppressed cell proliferation, migration and metastasis of Hep-2 cells *in vitro* and *in vivo*. The expression of miR-139 was decreased with the progression of primary to metastatic LSCC. Moreover, miR-139 expression level was inversely correlated with chemokine receptor 4 (CXCR4) levels in LSCC specimens. It was concluded that miR-139 inhibited proliferation and metastasis of LSCC through targeting CXCR4 [59]. MiR-203 was downregulated in the LSCC tissues and its expression level was inversely correlated with ASAP1. miR-203 inhibited cellular proliferation, invasion and induced apoptosis, G1 phase cell cycle arrest of Hep-2 cells *in vitro* through targeting ASAP1, a binding protein for a well-known oncogene, the Src family. In addition, miR-203 inhibited the growth of xenograft laryngeal tumors in mice. Therefore, the loss of miR-203 may play an important role in the progress of LSCC and act as a tumor suppressor in LSCC [60]. miR-206 inhibited LSCC cell proliferation, migration, invasion and tumorigenesis and increased apoptosis partly by inhibiting vascular endothelial growth factor (VEGF). Therefore, miR-206 may also function as a novel tumor suppressor worth further research [61]. A recent study showed that miR-299-3p was decreased in human laryngeal cancer cells. Overexpression of miR- 299-3p inhibited cellular proliferation by targeting the 3′-untranslated region of hTERT mRNA [62]. miR-370 was down-regulated in human LSCC tissues, inversely related with Forkhead Box ml (FoxM1). Furthermore, miR-370 decreased cell proliferation in Hep2 cells, indicating that miR-370 might function as a tumor suppressor in LSCC through downregulation of FoxM1 [63]. Recent study found that microRNA-519a inhibited cell growth in LSCC and led to cell cycle arrest via downregulating HuR gene expression. Therefore, mir-519a may function as a tumor suppressor by suppressing HuR expression [64]. miR- 519b- 3p, lower in laryngeal carcinoma tissues, inhibited Hep-2 cell proliferation and caused cell cycle arrest. HuR and cyclooxygenase-2 (COX-2) might be the potential target gene of miR-519b-3p. miR-874, downregulated in LSCCs, inhibited proliferation and induced of cell cycle arrest and apoptosis by targeting histone deacetylase 1 (HDAC1) [36] (Table 2).

**Table 2:** Summary of oncomirs and tumor suppressive miRs in laryngeal cancer

Upregulated (oncomiRs)		
miRs	Target	Final effects
miR-16	Zyxin	Migration and adehesion
miR-19a	TIMP2	Proliferation and apoptosis
miR-21	BTG2	Proliferation
miR-27a	PLK2	Proliferation and apoptosis
miR-106b	RUNX3	Proliferation and invasion
miR-129-5p	APC	Proliferation and migration, and cell cycle control
miR-155	SOCS1, STAT3	Proliferation and invasion
miR-196a	Not mentioned	Proliferation
miR-1297	PTEN	Proliferation and migration,
Downregulated (tumor suppressor miRs)		
miRs	Target	Functions
miR-1	FN1	Proliferation, migration and invasion
miR-24	S100A8	Proliferation and invasion
miR-30b	Not mentioned	Apoptosis
miR-34a	Survivin	Proliferation and cells cycle control
hsa-miR-34c	C-Met	Proliferation and invasion
miR-126	Camsap1	Aggregation
miR-139	CXCR4	Proliferation and metastasis
miR-203	ASAP1	Proliferation, invasion, apoptosis, cell cycle control
miR-206	VEGF	Proliferation, migration, invasion and apoptosis
miR-299–3p	hTERT	Proliferation
miR-370	Fox-M1	Proliferation
miR-519a	HuR, COX-2	Proliferation and cell cycle control
miR-874	HDAC1	Proliferation, cell cycle control and apoptosis

### Mechanisms of dysregulation of miRs in laryngeal cancer

Both genetic and epigenetic mechanisms can lead to miR dysregulation, which can be regulated via the introduction of single-nucleotide polymorphisms (SNP) into the miR sequence itself, or via aberrant DNA methylation and histone modification [65–71].

Dicer is an important cytoplasm enzyme in miR processing [72–74]. Dysregulation of Dicer or other enzymes in the miR biogenesis is hypothesized to be a common feature in tumors [75–79]. Reduced or increased Dicer expression in LSCC could lead to cancer deregulated miRs expressions, which play an important role in LSCC development. Expression of Dicer was higher in the LSCC, significantly correlated with the pTNM stage and tumor lymph node metastasis. In addition, Kaplan–Meier survival analyses revealed a strong association between tumor Dicer expression and the survival of the patients with LSCC [80]. Some miRs are silenced epigenetically by DNA hypermethylation. Among miRs in laryngeal cancer, the change in miR-137 involves aberrant hypermethylation [81]. In addition, Johnston et al. found pepsin from stomach might also contribute to deregulated miRs in laryngeal cancers. Pepsin altered the expression of 22 microRNAs in human head and neck cancers. Furthermore, pepsin could promote proliferation in both FaDu SCC cells and cultured normal laryngeal epithelial primary cells [82].

### MicroRNAs as early diagnostic biomarkers for laryngeal cancer

Accumulating studies have proved miRs as diagnostic biomarkers for laryngeal cancer. Since many miRs were investigated in a single study with small sample size, the results are inconclusive and need to be validated in independent studies.

Combination of miR-21 and miR-375 were overexpressed and underexpressed respectively in LSCC. Furthermore, the ratio of miR-21/miR-375 displayed a high predictive diagnosis potential, with a highly sensitivity (0.94) and specificity (0.94) for discriminating between in distinguishing tumor and normal tissue [44]. In a study to identify potential miR biomarkers for early diagnosis of larynx carcinoma, the hsa-miR-657-hsa-miR-1287 classifier, which was overexpressed and underexpressed respectively, demonstrated high sensitivity and specificity in distinguishing early larynx carcinoma from normal mucosa tissues. Based on this finding, the hsa-miR-657-hsa-miR-1287 classifier may serve as potential diagnostic biomarkers for early larynx carcinoma [38]. To investigate the alterations in plasma miRs in LSCC, Ayaz et al. found 17 miRs expressed specifically in plasma of LSCC patients and never seen before in plasma of any human subject. Furthermore, plasma miR-331-3p, 603, 1303, 660-5p and 212-3p were not expressed in healthy individuals and patients with any other diseases. Therefore, miR-331-3p, 603, 1303, 660-5p and 212-3p may serve as novel non-invasive biomarkers for diagnosis of LSCC [41]. Saito et al. found a laryngeal cancer-specific expression of miR-196a in both cancer and cancer stromal cells, suggesting its potential as diagnostic disease markers [39].

### MicroRNAs as prognostic biomarker for laryngeal cancer

Combined treatments with chemo and radiotherapy have substantially improved patient survival with LC. However, prognostic biomarkers for LC patients are limited [83, 84]. Wu et al. showed that higher expression of miR-19a in 83 LSCC patients correlated with shorter overall survival and with neck nodal metastasis, poor differentiation and advanced stage [43]. Yu et al demonstrated that miR-21 and miR-106b expressions were increased laryngeal cancer tissues and miR-375 was decreased. Moreover, miR-21 and miR-106b levels were inversely associated with differentiation and positively associated with lymph node metastasis and TNM stages [45]. Another study showed that high miR-21 or low miR- 375 expressions correlated with poorer LSCC prognoses [44, 45]. Both miR-126 and its target, Camsap1, were proved to related with the prognosis of LSCC patients [85]. Zhao et al. found higher miR-155 levels in advanced T stages and poor/moderate cell differentiation [51]. Shen *et al*. found that miR-34a expression levels were negatively correlated with histologic differentiation and were positively correlated with survival rate. miR-34a expressions could serve as an independent predictors for LSCC patient survival in a training set of 69 patients (*P* = 0.011) [86]. The expression of miR-139 was decreased with the progression from primary to metastatic LSCC, indicating its potential effect as a prognostic factor [59]. miR-203 expression was downregulated in LSCC tissues and Inverse correlation of miR-203 expression was found with poor differentiation, advanced clinical stages, T3–4 tumor grade, lymph node metastasis and decreased 5-year overall survival, hinting that miR-203 may be used as a predictive marker of survival rate in LSCC patients [60, 87]. ZHANG et al. found that miR-206 was lower in the LSCC tissues. Decreased expression of miR-206 was associated with the T grade, nodal metastasis and clinical stage of LSCC. Therefore, it was presumed that the loss of miR-206 might be associated with poor survival in LSCC patients [61]. Dicer, an important enzyme in the miR machinery was showed to be upregulated in the LSCC specimens. Moreover, the expression level of Dicer was associated with the pTNM stage and tumor lymph node metastasis. Kaplan– Meier survival analyses demonstrated a strong association between Dicer expression and the LSCC patient survival [80] (Table 3).

**Table 3:** Studies on prognostic functions of miRNAs in laryngeal cancer

Reference	Sample Size	miRNA	Prognosis
43	83	miR-19a	Poor survival, lymph node metastasis
45	Not mentioned	miR-21 and miR-106b	Poor survival, poor differentiation, lymph node metastasis and TNM stage
44, 45	46	miR-21	Poor prognosis
44, 45	46	miR-375	Good prognosis
51	63	miR-155	TNM stage and poor differentiation
86	69	miR-34a	Good survival
60, 87	56	miR-203	Good survival and good differentiation. Inversely correlated with advanced clinical stages, T3–4 tumor grade, lymph node metastasis
61	35	miR-206	Inversely correlated with T grade, nodal metastasis and clinical stage

### miRs associated with therapeutic resistance in laryngeal cancer

Multidrug resistance (MDR) is a serious problem of chemotherapy for laryngeal cancer. Microarray analysis showed that miRs (has-miR-210 and has-miR-923) were upregulated and five miRs (has-miR-93, has-miR-93-star, has-miR-424-star, has-miR-25-star, and has-miR-494) were downregulated in multidrug resistant Hep2/v cells compared with Hep-2 cells [88]. Paclitaxel, a widely used chemotherapy drug for advanced laryngeal cancer patients, altered the expression of 49 miRs, such as miR-31-star, miR-1264, miR-3150b-5p and miR-210 [34, 89]. Li et al showed that miR-30b significantly promoted p53-mediated tumor cell apoptosis in HEp-2 cells. Furthermore, miR-30b could obviously increase the anti-tumor and pro-apoptotic effect of Ad-p53 in HEp-2 implanted nude mice [56]. Upregulation or downregulation of a specific miR might result in therapeutic resistance to chemotherapy or radiotherapy, and it can be treated through modulating specific miRs. Therefore, new therapy may be developed with a combination of miR inhibitors or supplementation with chemotherapy or radiotherapy.

### Therapeutic potential of MicroRNAs in laryngeal cancer

Anticancer treatment may be achieved either by pharmacologic supplement of tumor suppressor miRs that are decreased in cancer tissue or inhibitors of oncomiRs that are increased. However, the delivery systems that introduce nucleic- acid-based drugs into cells are a challenge. For miR supplementation, extracellular administration of mature miR can't be recruited by the RISC complex is ineffective, therefore, it is an ineffective method [90]. Studies on the synthetic nucleic acid molecules that can be processed to mature miR in the cell are needed. For miR inhibition, antisense miR oligonucleotides (AMOs) are the most common miR inhibitors since it is ineffective to use siRNAs due to shortness of the strands [91]. For example, the oncogenic miR-129-5p could promote cell proliferation and migration, and induced cell cycle arrest and apoptosis in LSCC *in vitro* while the miR-129-5p antisense oligonucleotides (ASO) in BALB/c significantly inhibited of the growth of LSCC xenograft in mice [50]. Repeated subcutaneous injection of ASO-miR-19a lentivirus significantly inhibited the growth of LSCC xenograft tumors subcutaneously injected with Hep-2 cells [43].

## CONCLUSION

It is clear that miRs play significant roles in the pathogenesis of laryngeal cancer. Functional analyses of miRs may contribute to clinical applications in the near future. Several miRs have been identified to be consistently dysregulated in laryngeal cancer, including upregulation of miR-21 and miR-196a and downregulation of miR-203. Profiling using miR arrays may contribute to early diagnosis of larygeal cancer, as well as the detection of stage, and prognosis. Future studies with well-designed clinical outcome study of large sample size and consistent treatment are needed to investigate the role of miRs in the prognosis of laryngeal cancer. Functional studies have shown inhibition or forced expression of many miRs affects tumor cell survival and development *in vitro*. However, more *in vivo* studies are needed to prove their oncogenic or tumor suppressive effects. Future treatment of tumor may be achieved by inhibition of overexpressed oncogenic miRs or substitution of tumor suppressive miRs. Improvement in the stabilization and delivery methods for miRs is crucial to apply miR to future clinical therapy.

## References

[1] Yu X, Li Z, Liu J (2015). MiRNAs in primary cutaneous lymphomas. Cell Prolif.

[2] Yu X, Li Z (2014). MicroRNAs regulate vascular smooth muscle cell functions in atherosclerosis (review). Int J Mol Med.

[3] Gargalionis AN, Basdra EK (2013). Insights in microRNAs biology. Curr Top Med Chem.

[4] Hata A (2013). Functions of microRNAs in cardiovascular biology and disease. Annu Rev Physiol.

[5] Karp X, Ambros V (2005). Developmental biology. Encountering microRNAs in cell fate signaling. Science.

[6] Latronico MV, Catalucci D, Condorelli G (2007). Emerging role of microRNAs in cardiovascular biology. Circ Res.

[7] Weidhaas J (2010). Using microRNAs to understand cancer biology. Lancet Oncol.

[8] Li Z, Yu X, Shen J, Jiang Y (2015). MicroRNA dysregulation in uveal melanoma: a new player enters the game. Oncotarget.

[9] Li Z, Yu X, Shen J, Wu WK, Chan MT (2015). MicroRNA expression and its clinical implications in Ewing's sarcoma. Cell Prolif.

[10] Li Z, Yu X, Wang Y, Shen J, Wu WK (2014). By downregulating TIAM1 expression, microRNA-329 suppresses gastric cancer invasion and growth. Oncotarget.

[11] Bezan A, Gerger A, Pichler M (2014). MicroRNAs in testicular cancer: implications for pathogenesis, diagnosis, prognosis and therapy. Anticancer Res.

[12] Callegari E, Gramantieri L, Domenicali M, D'Abundo L, Sabbioni S (2015). MicroRNAs in liver cancer: a model for investigating pathogenesis and novel therapeutic approaches. Cell Death Differ.

[13] Liu C, Tang DG (2011). MicroRNA regulation of cancer stem cells. Cancer Res.

[14] Lovat F, Valeri N, Croce CM (2011). MicroRNAs in the pathogenesis of cancer. Semin Oncol.

[15] Mayne GC, Hussey DJ, Watson DI (2013). MicroRNAs and esophageal cancer–implications for pathogenesis and therapy. Curr Pharm Des.

[16] Mezzanzanica D, Bagnoli M, De Cecco L, Valeri B, Canevari S (2010). Role of microRNAs in ovarian cancer pathogenesis and potential clinical implications. Int J Biochem Cell Biol.

[17] Okayama H, Schetter AJ, Harris CC (2012). MicroRNAs and inflammation in the pathogenesis and progression of colon cancer. Dig Dis..

[18] Shah NR, Chen H (2014). MicroRNAs in pathogenesis of breast cancer: Implications in diagnosis and treatment. World J Clin Oncol.

[19] Sianou A, Galyfos G, Moragianni D, Andromidas P, Kaparos G (2015). The role of microRNAs in the pathogenesis of endometrial cancer: a systematic review. Arch Gynecol Obstet.

[20] Su X, Xing J, Wang Z, Chen L, Cui M (2013). microRNAs and ceRNAs: RNA networks in pathogenesis of cancer. Chin J Cancer Res.

[21] White NM, Yousef GM (2010). MicroRNAs: exploring a new dimension in the pathogenesis of kidney cancer. BMC Med.

[22] Burzynski NJ (1969). A statistical review of oral-pharyngeal and laryngeal cancer in Kentucky from 1962 through 1966. J Ky Dent Assoc.

[23] Galli J, Cammarota G, Volante M, De Corso E, Almadori G (2006). Laryngeal carcinoma and laryngo-pharyngeal reflux disease. Acta Otorhinolaryngol Ital.

[24] Tomeh C, Holsinger FC (2014). Laryngeal cancer. Curr Opin Otolaryngol Head Neck Surg.

[25] Voutilainen A, Tuovinen P (1962). Treatment of laryngeal cancer and its results. A review of 261 cases. Ann Chir Gynaecol Fenn.

[26] Hillman RE, Walsh MJ, Wolf GT, Fisher SG, Hong WK (1998). Functional outcomes following treatment for advanced laryngeal cancer. Part I--Voice preservation in advanced laryngeal cancer. Part II--Laryngectomy rehabilitation: the state of the art in the VA System. Research Speech-Language Pathologists. Department of Veterans Affairs Laryngeal Cancer Study Group. Ann Otol Rhinol Laryngol.

[27] Jenckel F, Knecht R (2013). State of the art in the treatment of laryngeal cancer. Anticancer Res.

[28] Fernandez-Hernando C, Suarez Y, Rayner KJ, Moore KJ (2011). MicroRNAs in lipid metabolism. Curr Opin Lipidol.

[29] Kanellopoulou C, Monticelli S (2008). A role for microRNAs in the development of the immune system and in the pathogenesis of cancer. Semin Cancer Biol.

[30] Donzelli S, Strano S, Blandino G (2014). microRNAs: short non-coding bullets of gain of function mutant p53 proteins. Oncoscience.

[31] Sanchez-Diaz PC, Hsiao TH, Zou Y, Sugalski AJ, Heim-Hall J (2014). In silico functional analyses and discovery of survival-associated microRNA signatures in pediatric osteosarcoma. Oncoscience.

[32] Hayes J, Thygesen H, Droop A, Hughes TA, Westhead D (2015). Prognostic microRNAs in high-grade glioma reveal a link to oligodendrocyte precursor differentiation. Oncoscience.

[33] Li Z, Yu X, Shen J, Jiang Y (2015). MicroRNA dysregulation in uveal melanoma: a new player enters the game. Oncotarget.

[34] Xu CZ, Xie J, Jin B, Chen XW, Sun ZF (2013). Gene and microRNA expression reveals sensitivity to paclitaxel in laryngeal cancer cell line. Int J Clin Exp Pathol.

[35] Cao P, Zhou L, Zhang J, Zheng F, Wang H (2013). Comprehensive expression profiling of microRNAs in laryngeal squamous cell carcinoma. Head Neck.

[36] Nohata N, Hanazawa T, Kinoshita T, Inamine A, Kikkawa N (2013). Tumour-suppressive microRNA-874 contributes to cell proliferation through targeting of histone deacetylase 1 in head and neck squamous cell carcinoma. Br J Cancer.

[37] Lu ZM, Lin YF, Jiang L, Chen LS, Luo XN (2014). Micro-ribonucleic acid expression profiling and bioinformatic target gene analyses in laryngeal carcinoma. Onco Targets Ther.

[38] Wang Y, Chen M, Tao Z, Hua Q, Chen S (2013). Identification of predictive biomarkers for early diagnosis of larynx carcinoma based on microRNA expression data. Cancer Genet.

[39] Saito K, Inagaki K, Kamimoto T, Ito Y, Sugita T (2013). MicroRNA-196a is a putative diagnostic biomarker and therapeutic target for laryngeal cancer. PLoS One.

[40] Tai J, Xiao X, Huang ZG, Yu ZK, Chen XH (2013). [MicroRNAs regulate epithelial-mesenchymal transition of supraglottic laryngeal cancer]. Zhonghua Er Bi Yan Hou Tou Jing Wai Ke Za Zhi.

[41] Ayaz L, Gorur A, Yaroglu HY, Ozcan C, Tamer L (2013). Differential expression of microRNAs in plasma of patients with laryngeal squamous cell carcinoma: potential early-detection markers for laryngeal squamous cell carcinoma. J Cancer Res Clin Oncol.

[42] Wu H, Liu T, Wang R, Tian S, Liu M (2011). MicroRNA-16 targets zyxin and promotes cell motility in human laryngeal carcinoma cell line HEp-2. IUBMB Life.

[43] Wu TY, Zhang TH, Qu LM, Feng JP, Tian LL (2014). MiR-19a is correlated with prognosis and apoptosis of laryngeal squamous cell carcinoma by regulating TIMP-2 expression. Int J Clin Exp Pathol.

[44] Hu A, Huang JJ, Xu WH, Jin XJ, Li JP (2014). miR-21 and miR-375 microRNAs as candidate diagnostic biomarkers in squamous cell carcinoma of the larynx: association with patient survival. Am J Transl Res.

[45] Yu X, Wu Y, Liu Y, Deng H, Shen Z (2014). miR-21, miR- 106b and miR-375 as novel potential biomarkers for laryngeal squamous cell carcinoma. Curr Pharm Biotechnol.

[46] Liu J, Lei DP, Jin T, Zhao XN, Li G (2011). Altered expression of miR-21 and PTEN in human laryngeal and hypopharyngeal squamous cell carcinomas. Asian Pac J Cancer Prev.

[47] Ren J, Zhu D, Liu M, Sun Y, Tian L (2010). Downregulation of miR-21 modulates Ras expression to promote apoptosis and suppress invasion of Laryngeal squamous cell carcinoma. Eur J Cancer.

[48] Tian Y, Fu S, Qiu GB, Xu ZM, Liu N (2014). MicroRNA-27a promotes proliferation and suppresses apoptosis by targeting PLK2 in laryngeal carcinoma. BMC Cancer.

[49] Xu Y, Wang K, Gao W, Zhang C, Huang F (2013). MicroRNA-106b regulates the tumor suppressor RUNX3 in laryngeal carcinoma cells. FEBS Lett.

[50] Li M, Tian L, Wang L, Yao H, Zhang J (2013). Down-regulation of miR-129-5p inhibits growth and induces apoptosis in laryngeal squamous cell carcinoma by targeting APC. PLoS One.

[51] Zhao XD, Zhang W, Liang HJ, Ji WY (2013). Overexpression of miR-155 promotes proliferation and invasion of human laryngeal squamous cell carcinoma via targeting SOCS1 and STAT3. PLoS One.

[52] Li X, Wang HL, Peng X, Zhou HF, Wang X (2012). miR-1297 mediates PTEN expression and contributes to cell progression in LSCC. Biochem Biophys Res Commun.

[53] Chen P, Wang BL, Pan BS, Guo W (2014). MiR-1297 regulates the growth, migration and invasion of colorectal cancer cells by targeting cyclo-oxygenase-2. Asian Pac J Cancer Prev.

[54] Wang F, Song G, Liu M, Li X, Tang H (2011). miRNA-1 targets fibronectin1 and suppresses the migration and invasion of the HEp2 laryngeal squamous carcinoma cell line. FEBS Lett.

[55] Guo Y, Fu W, Chen H, Shang C, Zhong M (2012). miR-24 functions as a tumor suppressor in Hep2 laryngeal carcinoma cells partly through down-regulation of the S100A8 protein. Oncol Rep.

[56] Li L, Wang B (2014). Overexpression of microRNA-30b improves adenovirus-mediated p53 cancer gene therapy for laryngeal carcinoma. Int J Mol Sci.

[57] Li W, Ma H, Sun J (2014). MicroRNA34a/c function as tumor suppressors in Hep2 laryngeal carcinoma cells and may reduce GALNT7 expression. Mol Med Rep.

[58] Cai KM, Bao XL, Kong XH, Jinag W, Mao MR (2010). Hsa-miR-34c suppresses growth and invasion of human laryngeal carcinoma cells via targeting c-Met. Int J Mol Med.

[59] Luo HN, Wang ZH, Sheng Y, Zhang Q, Yan J (2014). MiR-139 targets CXCR4 and inhibits the proliferation and metastasis of laryngeal squamous carcinoma cells. Med Oncol.

[60] Tian L, Li M, Ge J, Guo Y, Sun Y (2014). MiR-203 is downregulated in laryngeal squamous cell carcinoma and can suppress proliferation and induce apoptosis of tumours. Tumour Biol.

[61] Li S, Li Y, Wen Z, Kong F, Guan X (2014). microRNA-206 overexpression inhibits cellular proliferation and invasion of estrogen receptor alpha-positive ovarian cancer cells. Mol Med Rep.

[62] Li M, Chen SM, Chen C, Zhang ZX, Dai MY (2015). microRNA2993p inhibits laryngeal cancer cell growth by targeting human telomerase reverse transcriptase mRNA. Mol Med Rep.

[63] Yungang W, Xiaoyu L, Pang T, Wenming L, Pan X (2014). miR-370 targeted FoxM1 functions as a tumor suppressor in laryngeal squamous cell carcinoma (LSCC). Biomed Pharmacother.

[64] Shen Z, Zhan G, Deng H, Ren Y, Ye D (2013). MicroRNA-519a demonstrates significant tumour suppressive activity in laryngeal squamous cells by targeting anti-carcinoma HuR gene. J Laryngol Otol.

[65] Chen WS, Leung CM, Pan HW, Hu LY, Li SC (2012). Silencing of miR-1-1 and miR-133a-2 cluster expression by DNA hypermethylation in colorectal cancer. Oncol Rep.

[66] Diao Y, Guo X, Jiang L, Wang G, Zhang C (2014). miR-203, a tumor suppressor frequently down-regulated by promoter hypermethylation in rhabdomyosarcoma. J Biol Chem.

[67] Huang YW, Kuo CT, Chen JH, Goodfellow PJ, Huang TH (2014). Hypermethylation of miR-203 in endometrial carcinomas. Gynecol Oncol.

[68] Javeri A, Ghaffarpour M, Taha MF, Houshmand M (2013). Downregulation of miR-34a in breast tumors is not associated with either p53 mutations or promoter hypermethylation while it correlates with metastasis. Med Oncol.

[69] Tsai KW, Liao YL, Wu CW, Hu LY, Li SC (2011). Aberrant hypermethylation of miR-9 genes in gastric cancer. Epigenetics.

[70] Tsuruta T, Kozaki K, Uesugi A, Furuta M, Hirasawa A (2011). miR-152 is a tumor suppressor microRNA that is silenced by DNA hypermethylation in endometrial cancer. Cancer Res.

[71] Xu L, Wang F, Xu XF, Mo WH, Xia YJ (2011). Down-regulation of miR-212 expression by DNA hypermethylation in human gastric cancer cells. Med Oncol.

[72] Andl T, Murchison EP, Liu F, Zhang Y, Yunta-Gonzalez M (2006). The miRNA-processing enzyme dicer is essential for the morphogenesis and maintenance of hair follicles. Curr Biol.

[73] Frederikse PH, Donnelly R, Partyka LM (2006). miRNA and Dicer in the mammalian lens: expression of brain-specific miRNAs in the lens. Histochem Cell Biol.

[74] Lee YS, Nakahara K, Pham JW, Kim K, He Z (2004). Distinct roles for Drosophila Dicer-1 and Dicer-2 in the siRNA/miRNA silencing pathways. Cell.

[75] Chen JC, Su YH, Chiu CF, Chang YW, Yu YH (2014). Suppression of Dicer increases sensitivity to gefitinib in human lung cancer cells. Ann Surg Oncol.

[76] Jafari N, Dogaheh HP, Bohlooli S, Oyong GG, Shirzad Z (2013). Expression levels of microRNA machinery components Drosha, Dicer and DGCR8 in human (AGS, HepG2, and KEYSE-30) cancer cell lines. Int J Clin Exp Med.

[77] Faggad A, Kasajima A, Weichert W, Stenzinger A, Elwali NE (2012). Down-regulation of the microRNA processing enzyme Dicer is a prognostic factor in human colorectal cancer. Histopathology.

[78] Selever J, Gu G, Lewis MT, Beyer A, Herynk MH (2011). Dicer-mediated upregulation of BCRP confers tamoxifen resistance in human breast cancer cells. Clin Cancer Res.

[79] Ting AH, Suzuki H, Cope L, Schuebel KE, Lee BH (2008). A requirement for DICER to maintain full promoter CpG island hypermethylation in human cancer cells. Cancer Res.

[80] Gao C, Li X, Tong B, Wu K, Liu Y (2014). Up-regulated expression of Dicer reveals poor prognosis in laryngeal squamous cell carcinoma. Acta Otolaryngol.

[81] Langevin SM, Stone RA, Bunker CH, Lyons-Weiler MA, LaFramboise WA (2011). MicroRNA-137 promoter methylation is associated with poorer overall survival in patients with squamous cell carcinoma of the head and neck. Cancer.

[82] Johnston N, Yan JC, Hoekzema CR, Samuels TL, Stoner GD (2012). Pepsin promotes proliferation of laryngeal and pharyngeal epithelial cells. Laryngoscope.

[83] Choe MH, Min JW, Jeon HB, Cho DH, Oh JS (2015). ERp57 modulates STAT3 activity in radioresistant laryngeal cancer cells and serves as a prognostic marker for laryngeal cancer. Oncotarget.

[84] de Miguel-Luken MJ, Chaves-Conde M, de Miguel-Luken V, Munoz-Galvan S, Lopez-Guerra JL (2015). MAP17 (PDZKIP1) as a novel prognostic biomarker for laryngeal cancer. Oncotarget.

[85] Sun X, Wang ZM, Song Y, Tai XH, Ji WY (2014). MicroRNA-126 modulates the tumor microenvironment by targeting calmodulin-regulated spectrin-associated protein 1 (Camsap1). Int J Oncol.

[86] Shen Z, Zhan G, Ye D, Ren Y, Cheng L (2012). MicroRNA-34a affects the occurrence of laryngeal squamous cell carcinoma by targeting the antiapoptotic gene survivin. Med Oncol.

[87] Bian K, Fan J, Zhang X, Yang XW, Zhu HY (2012). MicroRNA-203 leads to G1 phase cell cycle arrest in laryngeal carcinoma cells by directly targeting survivin. FEBS Lett.

[88] Yin W, Wang P, Wang X, Song W, Cui X (2013). Identification of microRNAs and mRNAs associated with multidrug resistance of human laryngeal cancer Hep-2 cells. Braz J Med Biol Res.

[89] Xu CZ, Shi RJ, Chen D, Sun YY, Wu QW (2013). Potential biomarkers for paclitaxel sensitivity in hypopharynx cancer cell. Int J Clin Exp Pathol.

[90] Banno K, Iida M, Yanokura M, Kisu I, Iwata T (2014). MicroRNA in cervical cancer: OncomiRs and tumor suppressor miRs in diagnosis and treatment. ScientificWorldJournal.

[91] Weiler J, Hunziker J, Hall J (2006). Anti-miRNA oligonucleotides (AMOs): ammunition to target miRNAs implicated in human disease?. Gene Ther.

